# A Peculiar Polyp: Malakoplakia Found in Colon Polyp on Surveillance Colonoscopy

**DOI:** 10.51894/001c.166038

**Published:** 2026-08-01

**Authors:** Brandon Wiggins, Mark Rigby, J. Michael Sullivan, Lina Ataya, Sandeep Bhangu, Derek Thigpin

**Affiliations:** 1 Henry Ford - Genesys

## 132

### Introduction

Malakoplakia is an uncommon inflammatory condition that usually affects the urinary tract in immunocompromised, transplant recipients. The presentation of the disease as a colon polyp is exceedingly rare. This case, discovered incidentally during surveillance colonoscopy, adds to the limited literature on this unusual presentation.

### Case

An 81 year old female with ESRD status post kidney transplant on mycophenolate, prednisone and tacrolimus presented for surveillance colonoscopy. She was found to have an atypical cecal polyp with polypectomy histology revealing lamina propria expansion with macrophages possessing abundant eosinophilic cytoplasm and Michaelis-Guttman bodies, consistent with malakoplakia. Stains and stool studies were negative for infection.

The patient was referred to transplant infectious disease and had the diagnosis confirmed with tertiary care pathology. The patient was started on levofloxacin 750 mg daily with no subsequent symptoms or complications.

### Discussion

Malakoplakia is primarily associated with patients that are immunocompromised and has a predilection for the genitourinary tract. However, in rare cases they have been found in the gastrointestinal tract. This condition is primarily associated with incomplete bactericidal function of the macrophages, resulting in incomplete eradication of bacteria. The abnormal macrophages pathognomonic for malakoplakia are called Michaelis-Gutmann bodies, which stain positively on PAS and iron stains and can be easily confused with malignancy.. Treatment is imperative, as most patients are immunocompromised and is typically achieved with indefinite antibiotics. Fluoroquinolones are very useful due to macrophage bioavailability. If failure to identify and treat appropriately, patients may suffer graft failure, other organ failure and death due to perforation or hemorrhage.

### Conclusion

This case illustrates a rare colonic manifestation of malakoplakia in an immunosuppressed transplant recipient, emphasizing the importance of recognizing its characteristic Michaelis–Guttman bodies to avoid misdiagnosis. Given its potential to mimic malignancy and its association with impaired macrophage function, clinicians should maintain a high index of suspicion when encountering atypical colonic lesions in immunocompromised patients. Prompt identification and targeted antibiotic therapy can prevent unnecessary complications and death.

